# Cognitive Biases and Heuristics in Surgical Settings: A Systematic Review

**DOI:** 10.1097/SLA.0000000000006736

**Published:** 2025-04-21

**Authors:** Holly Aylmore, Srishti Agarwal, Hani J. Marcus, Anand S. Pandit

**Affiliations:** * UCL Queen Square Institute of Neurology, University College London, London, UK; † UCL Medical School, University College London, London, UK; ‡ Victor Horsley Department of Neurosurgery, National Hospital for Neurology and Neurosurgery, London, UK; § Wellcome/EPSRC Centre for Interventional and Surgical Sciences, University College London, London, UK; ∥ High-Dimensional Neurology Group, UCL Queen Square Institute of Neurology, University College London, London, UK

**Keywords:** cognitive bias, heuristics, surgery, surgical error, surgical safety

## Abstract

**Objective:**

To identify cognitive biases and heuristics experienced by surgeons in operative settings and the impact these biases and heuristics have on patient care.

**Background:**

Cognitive biases and heuristics are systematic errors in thinking that can affect clinical decisions. Both are noted in surgical settings and are a risk to patient safety.

**Methods:**

This review was conducted in accordance with Preferred Reporting Items for Systematic Reviews and Meta-Analyses guidelines and PROSPERO registered (CRD42023432099). Five major databases were searched from inception to August 28, 2022, with an updated search on January 27, 2024. Original primary research studies in English were included, with relevant risk of bias tools employed for each study.

**Results:**

Twenty-one papers were included. Thirty-eight biases were identified across 6 experiments, 5 analyses, and 10 survey studies. Confirmation bias, anchoring, risk aversion, and overconfidence bias were the most represented. Risk of bias was moderate across most studies. Cognitive biases and heuristics were found to influence surgical outcomes and 6 studies cited a negative impact on patient care, with one associating biases with fatal outcomes.

**Conclusions:**

Biases and heuristics contribute to surgical errors and never events, and will continue to do so until they are recognised and addressed. Implementing debiasing strategies, such as mindfulness training and deliberate reflection, was found to reduce surgical errors in 2 studies. This review highlights the need for experimental studies, which are essential for understanding how and why biases lead to negative outcomes and for evaluating further debiasing interventions. We propose directions for future research and system changes.

Despite the considerable knowledge and expertise possessed by surgeons, their clinical judgment can be affected by cognitive biases and heuristics.^1^ Cognitive biases are defined as systematic errors in thinking that deviate from rationality and influence decisions and judgments.^2^ They often arise from mental shortcuts known as heuristics, which are employed to process information rapidly and efficiently.^2^ However, while these shortcuts can in many circumstances prove advantageous, they may also lead to errors—particularly in stressful and demanding situations.

Cognitive biases can contribute toward surgical complications and never events, serious patient safety incidents that are entirely preventable. In a quality improvement study of 5256 surgical operations, during which 182 adverse events were identified, 38 events (21%) were attributed to cognitive biases.^3^ Such biases and heuristics are associated with postoperative complications,^4^ morbidity,^5,6^ and mortality.^7^

In the last decade, 2 systematic reviews of decision making in surgery identified a need to assess the prevalence of cognitive biases and their impact on medical errors and patient outcomes.^8,9^ In addition, a review in neurosurgery found a lack of training tools that would aid in the reduction of biases.^10^ Despite this, the incidence and prevalence of such biases in surgical settings remain underinvestigated.

To reduce the risk of complications and never events caused by biases, it is crucial to understand how these biases affect surgery and whether debiasing tools can mitigate risks and improve patient outcomes. This review aims to identify cognitive biases in surgical settings and examine their impact on surgical errors, never events, and patient outcomes.

## METHODS

This review was conducted in accordance with the Preferred Reporting Items for Systematic Reviews and Meta-Analyses (PRISMA) guidelines.^11^ A completed PRIMSA Checklist is available in Supplemental Digital Content (Supplemental Digital Content 1, http://links.lww.com/SLA/F474). The review protocol was registered on the PROSPERO database (CRD42023432099).

### Search Strategy

A search of 5 databases, Cochrane Controlled Register of Trials (CENTRAL), Embase (via Ovid), MEDLINE (via Ovid), Web of Science, and SCOPUS was performed on August 28, 2022. An updated search to identify additional papers was run on January 27, 2024. All databases were searched from their inception, and no filters or limitations were applied. Medical Subject Headings terms and keyword searches for “surgery,” “cognitive bias,” and “heuristics” and their synonyms were combined. Full search strategies were reviewed by our institute librarian and are available in Supplemental Digital Content (Supplemental Digital Content 1, http://links.lww.com/SLA/F474).

### Eligibility Criteria

Inclusion criteria were: original primary research studies, published in English, that investigated cognitive biases and/or heuristics with a clear assessment of the bias(es) and/or heuristic(s) in the study design, conducted on surgeons in surgical settings, including surveys of surgical vignettes. Exclusion criteria were: single case reports, secondary research studies such as reviews, letters to the editor, commentaries, and opinion pieces, studies that explained results or attributed findings to cognitive biases and/or heuristics but did not investigate them as part of the study design, studies of social biases, studies not conducted in surgeons and not in surgical settings, including surveys not related to surgeries. Experts in the field were consulted for their opinion on any papers that met eligibility criteria but were not identified through the search.

### Study Selection

Results of the final searches in all databases were combined, and after the removal of duplicates, an initial screening of the title and abstract of each article using the inclusion criteria was performed. Articles included at this stage were then full-text screened to ensure they were eligible. All stages of screening were carried out independently by 2 reviewers (H.A. and S.A.) using Rayyan,^12^ and any discrepancies between the 2 screenings were discussed and resolved with a third reviewer (A.S.P.). Full details of the data extracted from each paper are available in Supplemental Digital Content (Supplemental Digital Content 1, http://links.lww.com/SLA/F474).

### Quality Assessment

Four tools were used to assess the risk of bias depending on the study design of the included papers. Randomized control trials were evaluated using the Cochrane Risk-Of-Bias tool for randomized trials,^13^ the Methodological Index for Non-Randomized Studies tool^14^ for nonrandomized and observational surgical studies, the Newcastle-Ottawa Scale tool^15^ for cohort studies, and survey studies were evaluated using the Critical Appraisal of a Survey tool.^16^ Given the use of multiple tools, final risk of bias scores for each were identified as low risk if scored above 75%, moderate risk if scored between 50% and 75%, and high risk if scored below 50%.

### Data Synthesis

Narrative synthesis and summary statistics were generated for identified cognitive biases and heuristics. Due to the heterogeneity of reporting within included studies, a planned meta-analysis of odds ratios to calculate a single estimate of the association between the presence of a bias and the incidence of surgical complications could not be performed.

## RESULTS

### Identification of Eligible Studies

**FIGURE 1:**
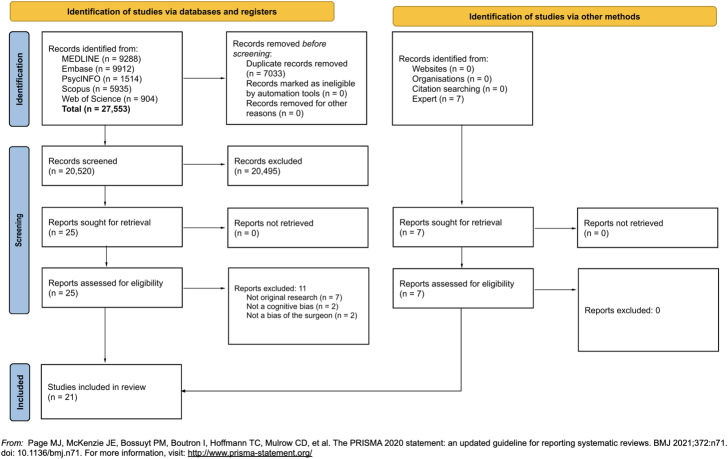
PRISMA 2020 flow diagram for new systematic reviews, which included searches of databases, registers, and other sources. PRISMA indicates Preferred Reporting Items for Systematic Reviews and Meta-Analyses.

A total of 27,553 articles were identified from the search (Fig. 1). After the removal of duplicates, 20,520 titles and abstracts were screened. Following a full text screen of the resulting 25 articles, 14 were identified that met the inclusion criteria. An additional 7 papers were identified in consultation with experts, giving a total of 21 papers that were included in the final analysis.

### Quality Assessment

**FIGURE 2:**
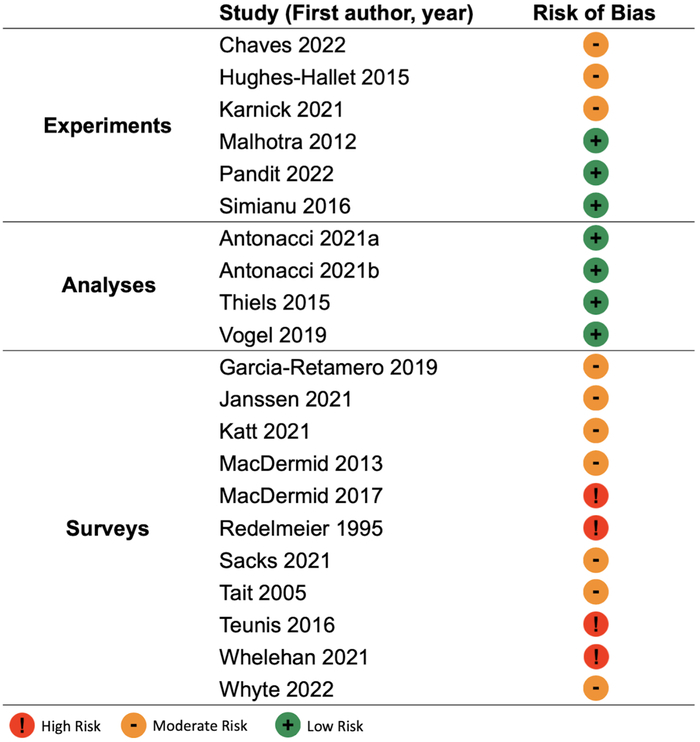
Risk of bias analysis.

Risk of bias analyses indicated moderate risk across studies, with 7 identified as low risk (33%), 10 as moderate risk (48%), and 4 studies being deemed as high risk (19%) due to factors including selection bias and the use of nonvalidated surveys (Fig. 2).

### Study Characteristics

The majority of included articles (Supplemental Digital Content 1, http://links.lww.com/SLA/F474) were surveys (11/21, 52%). The remaining studies were experiments (6/21, 29%) and retrospective or prospective analyses of data, such as incidence rates (4/21, 19%). A range of specialities were represented across studies, with general surgery and pan-speciality articles most common (5/21, 23%), followed by orthopedics (4/21, 19%), rectal, colorectal, and neurosurgery (2/21, 9%), and laparoscopic and unspecified specialities (1/21, 4%). Funding information provided by study authors is available in Supplemental Digital Content (Supplemental Digital Content 1, http://links.lww.com/SLA/F474).

**FIGURE 3:**
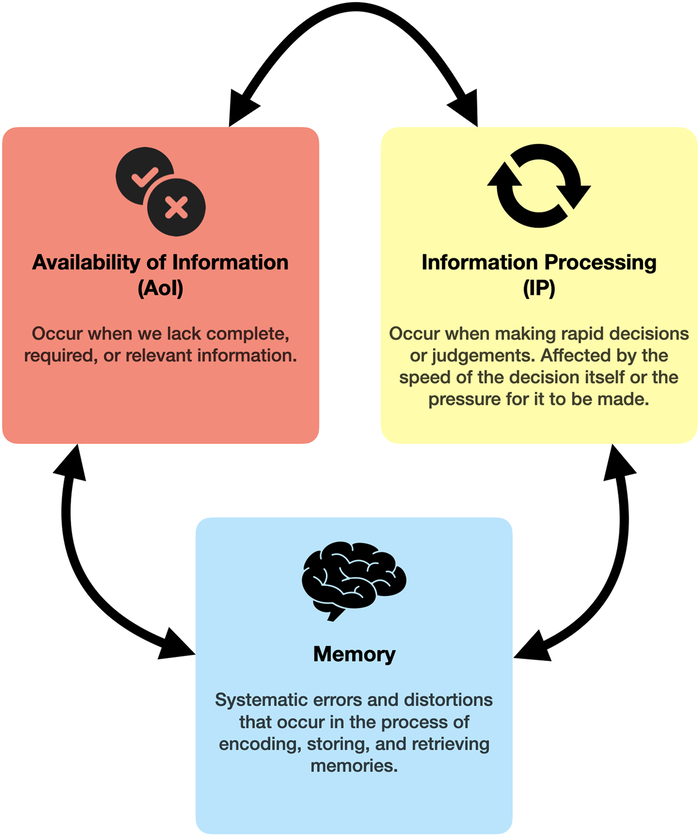
Bias categories and their definitions.

Across the 21 studies, 38 biases were identified. Biases were identified using 5 different modalities across studies: by objective measures (8/21, 38%), by author developed surveys (6/21, 29%), by surgeons and/or evaluators trained to identify biases (3/21, 14%), by validated surveys (3/21, 14%), or by a validated tool (1/21, 5%). There have been several attempts to categorize and form taxonomies of known cognitive biases and heuristics, including a task-based taxonomy,^17^ memory model,^18^ and noise in information model,^19^ but none have yet achieved broad acceptance. No categorization or taxonomy of cognitive biases and heuristics that encompasses all identified biases and heuristics in this review has been formulated. In the absence of an established taxonomy, here, we categorize biases and heuristics based on the way in which the error in thinking or mental shortcut mishandles information because of either (1) its availability, (2) how it is processed, or (3) how it is remembered (Fig. 3).

### Key Results

For each identified bias, definitions and an example of the bias in a surgical setting are available in Supplemental Digital Content (Supplemental Digital Content 1, http://links.lww.com/SLA/F474).

#### Availability of Information

Cognitive biases of availability of information occur when we lack complete, required, or relevant information, leading to inaccurate judgments and flawed reasoning. 12 biases concerning the availability of information were identified across 5 studies.^20–23^

Anchoring was the second most commonly identified bias in this review. Identified in 5 studies, the presence of anchoring was associated with management and therapeutic errors, severe complications, and an increased likelihood of patient harm in an analysis of 655 general surgery cases.^20^ Notably, it was associated with fatal outcomes in a study of 736 general surgery cases.^21^ Anchoring was also identified in a survey of 196 orthopedic surgeons consisting of 3 surgical vignettes^22^ and in one case of anastomotic failure.^24^ However, a survey study of 73 surgeons from the Royal College of Surgeons in Ireland found that incorporating a “hook”—additional clinically relevant information designed to evoke a bias, in this case anchoring—into described clinical cases did not significantly change decision-making behavior.^23^

Overconfidence bias, identified in 4 studies. It was found to be the most commonly identified bias in cases with complications, the most associated with probability of harm, management errors, and therapeutic errors, to be associated with serious complications in one prospective cohort analysis.^20^ It was frequently associated with fatal outcomes and to have a statistically significant association with management errors in another.^21^ Overconfidence bias was also identified in a case of anastomotic failure in colorectal surgery,^24^ and in a survey of 242 orthopedic surgeons in which 83% considered themselves to be above average diagnosticians and none regarded themselves as below average.^25^

Ascertainment bias, observed in 2 analyses of general surgery cases, had mixed results. In one study, it was the most commonly identified bias in cases with complications,^21^ but in another was not found to have a statistically significant probability of causing severe harm.^20^ Other biases, including order effects, psych-out error, search satisfying, triage cueing, the unpacking principle, and yin-yang out, were also not found to have a statistically significant probability of causing severe harm in the same study.^20^ There were also mixed findings for premature closure of decision making, which was not associated with severe harm in one study, but was the most commonly associated with an increase in the incidence of management errors in another.^21^

In a structured interview study, availability bias was identified in one case of anastomotic failure in colorectal surgery,^24^ but in a clinical case survey of 73 surgeons in which additional clinically relevant information designed to evoke availability bias, known as a “hook,” was placed, no significant change to decision making behavior was found.^23^ The final availability of information bias found in this review was base rate neglect, which was identified in 43% of 196 orthopedic surgeons in a survey of 3 surgical vignettes.^22^

#### Information Processing

Cognitive biases of information processing occur when making rapid decisions or judgments. Decision making is affected by the speed of the decision itself, either too fast or too slow, or the need for a quick decision to be made. These biases are often the result of the brain’s tendency to rely on efficient and automatic processes. Nineteen biases concerning information processing were identified across 16 studies,^20–22,24–36^ 3 of which found links to patient harm.^20,21,24^

Risk aversion was the most commonly identified bias in this group, found in 4 survey studies where it influenced surgeons’ clinical decision making. A survey of 110 colorectal surgeons highlighted risk aversion as an unrecognized factor in the decision to create a stoma after anterior resection.^29^ Similarly, surgeons over the age of 50 who were members of the Association of Coloproctology Great Britain and Ireland were found to be less risk-averse, making them less likely to create a defunctioning stoma after a range of anterior resections.^30^ A clinical vignette survey of 1769 general surgeons found risk averse surgeons were less likely to recommend an operation than risk seeking surgeons for identical pathologies,^34^ and risk aversion was identified in 76.47% of 53 surveyed breast surgeons.^36^

Found to be linked with direct patient harm, commission bias was identified in 3 analysis studies. It was associated with a >20% increased incidence of severely harmed patients compared with minor harmed patients,^20^ correlated with a statistically significant increase in the incidence of management errors,^21^ and was identified intraoperatively in a case of colorectal resection in which a patient suffered an anastomotic failure.^24^ Omission bias was also identified in these 3 studies, being correlated to a statistically significant increase in management errors^21^ and identified in 2 cases of anastomotic failure,^24^ though the probability of omission bias causing severe harm to patients in 655 general surgery cases with complications was not statistically significant.^20^

Sunk cost fallacy had similar mixed findings relating to patient harm. It was identified in a single case of colorectal resection in which a patient suffered an anastomotic failure,^24^ but not associated with a statistically significant probability of causing severe harm in general surgery cases.^20^ In the same study, diagnosis momentum was associated with a >20% increased incidence in severely harmed patients,^20^ and correlated to a statistically significant increase in incidence in management errors in another analysis of general surgery cases.^21^ Other biases, including aggregate bias, outcome bias, suttons slip, and vertical line failure were identified but not associated with more serious complications or a higher probability of harm.^20^

Two experimental studies identified inattention blindness. In a randomized controlled study of 73 surgeons, high levels of inattention blindness outside of the surgeons’ tunnel of focus were found, with increased cognitive load increasing inattention.^27^ Furthermore, in a prospective controlled pilot study, neurosurgeons who completed an 8 week mindfulness based intervention made significantly less errors on a task measuring inattention blindness in comparison to a control group.^32^

Perceptual bias was identified in 20 novice and 11 experienced surgeons completing a simulated incision-making task, with both novice and experienced surgeons perceiving the incision to be smaller than it was.^31^ Surgical experience was found to be protective against this underestimation as the task became more visually challenging.^31^ In a prospective cohort study of 282 colorectal surgeons examining the use of preventative leak testing before and after operations with anastomotic leaks, 36% of surgeons increased their leak testing by 5% or more after they experienced leaks in cases where anastomosis was not tested, demonstrating recency bias.^35^

A retrospective analysis of 9 orthopedic surgeons’ self-assessment of the time needed to complete operative procedures found optimism bias and planning fallacy, with surgeons underestimating the total room time required by 17.3% (*P* = 0.034).^28^ Further biases of information processing were found in 3 survey studies. Denominator neglect was found in one survey of 300 surgeons in which surgeons were misled and biased by the information formats used to communicate risk in scientific literature.^26^ Framing was found to have a measurable effect in 5 surgical vignettes in a survey of 196 orthopedic surgeons.^22^ In a survey including neurosurgeons, the framing of treatment options affected surgeons’ choice, with an increased tendency to choose to prioritize operating on a clinically distinctive patient instead of 2 similar patients.^33^ Finally, a survey of breast surgeons found breast reconstruction preference changes, such as choices concerning the type of procedure and type of implant, when procedures were framed positively or negatively.^36^ In the same survey of 53 breast surgeons, endowment effect, herding bias, and illusion of control were found in 47.06%, 76.47%, and 41.48% of surgeons, respectively.

#### Memory

Cognitive biases of memory refer to systematic errors and distortions that can occur in the process of encoding, storing, and retrieving memories. Such biases can affect recollection of past events and information, leading to inaccuracies. Seven biases concerning memory were identified across 9 studies.^20–23,36–40^

Confirmation bias was the most commonly identified bias (7 studies). In a controlled experimental study, it was observed in 26.3% of initial diagnoses and 19.5% of final diagnoses when orthopedic surgeons were asked to solve clinical cases with an incorrect referral diagnosis.^37^ In 2 analyses, it was associated with management and therapeutic errors, but did not have a statistically significant probability of causing severe harm to patients,^20^ and was not commonly associated with fatal complications.^21^ In another prospective analysis study of 1.5 million procedures, confirmation bias was the most common perceptual error, making up 82% of all identified errors, and contributed to never events.^40^ It was further identified in 3 survey studies, in a clinical vignette survey of 196 orthopedic surgeons,^22^ in a survey of 53 breast surgeons 31.37% displayed confirmation bias,^36^ though in a survey of 73 surgeons from the Royal College of Surgeons in Ireland who were presented with a scenario intended to evoke confirmation bias, the presence of this “hook” did not lead to the bias.^23^

Self-evaluation bias was found in an experiment and survey study in first-year general surgeons who self-assessed their own performance on a battery of technical skills. When this performance was compared with their actual performance, the actual scores and self assessment scores were positively correlated with surgeons generally underestimating their performance, and those who performed above the cohort average assessed themselves more negatively.^38^ In 2 analyses of general surgery cases, hindsight bias was found to be one of the most commonly identified biases,^21^ but did not have a statistically significant probability of causing severe harm to patients.^20^ Posterior probability error and visceral bias were also identified in this study, though they too did not have a statistically significant probability of causing severe harm.^20^

Two further biases of memory were identified in survey studies. Self-serving attributional bias was found in a survey of 17 neurosurgeons and 23 orthopedic surgeons, with surgeons more likely to attribute failed surgery, relative to successful surgery, to patient psychological factors than their own performance,^39^ and the representation heuristic was found in 67.3% of 53 surveyed breast surgeons.^36^

## DISCUSSION

### Summary of Results

This review identified 38 cognitive biases and heuristics present in surgical settings, finding that even in the current best standard of care, biases and heuristics contribute to surgical errors, never events, and negative patient outcomes. However, there is potential to reduce both their incidence and effect on patients by implementing debiasing strategies.

Cognitive biases were directly associated with complications and patient harm in 4 studies,^20,21,24,40^ with overconfidence bias and anchoring being associated with fatal outcomes.^21^ The identified biases and heuristics are common and broad in variety, they are not specialty-specific, and are associated with the occurrence of never events^40^ and surgical complications.^21,24^ Debiasing strategies, such as diagnosis reviews, were identified in one study^20^ and a mindfulness-based strategy successfully implemented in another.^32^

### Quality of Included Studies

Though the findings of this review show that cognitive biases and heuristics contribute to negative surgical outcomes, the risk of bias analysis mitigates the strength of these findings. Two biases, confirmation bias and anchoring, were identified in 7 and 5 studies, respectively, and so are overrepresented in comparison to other identified biases in this review. The current evidence base is of low quality, with the study design of 11 studies (52%) employing nonvalidated surveys and surveys of simulated clinical vignettes, and, therefore, we cannot conclude that the biases and heuristics identified in these studies directly impact patients. However, despite this limitation, 3 studies (14%) were retrospective analyses of real patient cases^20,21,40^ and all associated the occurrence of cognitive biases and heuristics with patient harm.

The way in which cognitive biases and heuristics were identified in all studies, whether survey, analysis, or experiment, also reduces the quality of evidence. Biases and heuristics were often attributed to cases by authors with no study examining the cause of the identified bias or heuristic directly. In addition, as 7 studies^20–24,28,36^ (33%) identified multiple cognitive biases in surveys or analyses, it is possible that one bias or heuristic does not cause one type of error or never event, and that negative outcomes may be the result of the cumulative effect of multiple biases and include factors outside of the bias or heuristic itself.

### Implications of Results and Context

Taken together, the studies included in this review indicate an association between the occurrence of cognitive biases, heuristics, and negative patient outcomes. This association has significant implications for patient safety as the identification of biases and heuristics during surgery is currently uncommon in clinical practice, we do not know the underlying cause of these biases, and few studies have investigated ways to mitigate their impact.

Simply identifying and acknowledging biases is insufficient to fully address their impact. While it is useful to know which biases and heuristics are the most common in surgical settings as a starting point to improve awareness of their potential impact, the field must work towards studies that provide higher levels of evidence. Future studies need to employ objective measures of identifying biases and heuristics in both simulated studies and analyses of real cases to fully quantify and understand the cause and extent of the problem.

The results of this review are in line with a recent systematic review^41^ in which cognitive biases were identified in surgeons and found to negatively impact patient outcomes. However, among several key methodological differences, this review focused on nonsurgical settings, included medical students, and included studies that did not directly investigate biases and heuristics as part of their methodology but attributed the results to them.

### Debiasing

A key area of future research will concern debiasing strategies that work to prevent the incidence of cognitive biases and heuristics and, therefore, the negative patient outcomes and never events that can result from them. Given the number of different cognitive biases and heuristics found in surgical settings, identifying psychological mechanisms that underlie multiple biases would allow debiasing strategies to target more than one bias at a time.

**FIGURE 4:**
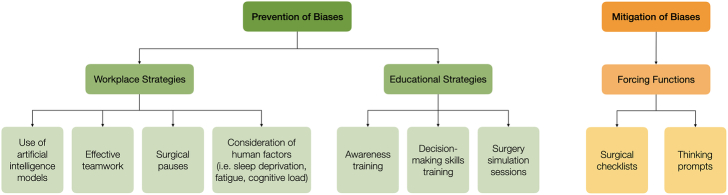
Debiasing strategies, modeled on strategies detailed in the study by Fargen and Friedman.^42^

Strategies for debiasing (Fig. 4) include metacognition, prompting, and reflection^43,44^ and have been previously grouped into a 3-part framework.^42^ Workplace strategies, such as the building and maintenance of effective teams, have been found to reduce the impact of anchoring and confirmation bias,^45^ and the integration of artificial intelligence (AI) models into the workplace has been shown to augment decision making and mitigate modifiable risks.^46^

While no studies included in this review assessed the role of AI in mitigating cognitive biases, a review of AI in surgical decision-making identified machine, deep, and reinforcement learning models that improved surgical decision-making using intraoperative time-series data, thus decreasing the chance of human cognitive bias.^46^ AI-assisted surgical coaching models have also been shown to improve surgical performance^47^ and could be adapted to include prompts to prevent cognitive biases. Despite this promise, it should be noted that as AI aims to mimic human learning, problem-solving, and decision-making, the field has to be careful to ensure that human cognitive biases are not integrated into the models themselves. Some models have been found to display human-like cognitive biases when recommending treatment, including surgery,^48^ and humans who were assisted by an intentionally biased AI system when performing a medical diagnostic task then demonstrated that bias themselves when completing the task without assistance.^49^

Forcing functions, such as checklists, have been successfully implemented to resolve errors in surgery such as the prevention of surgical site infections,^50^ and though there is a need for such checklists to be validated and standardized^51^ they could be applied to address biases and heuristics.

Educational debiasing strategies can be employed during surgical training, though this practice is currently ad hoc and institution-dependent. Research has noted that a common challenge in training is correcting the belief that good decision making can only be learned through personal experience,^52^ a belief that must be changed in order for the effect of biases and heuristics to be understood and acknowledged. Early promotion of the message that cognitive biases affect all surgeons, and are not the result of an individual’s personal fault or lack of skill, will be key. The Royal College of Surgeons of England has developed a free online training module exploring how cognitive biases can lead to errors in judgment and decision making during surgical training,^53^ and a 2016 World Health Organization report on diagnostic error directly calls for explicit training on cognitive biases and heuristics.^54^

**FIGURE 5:**
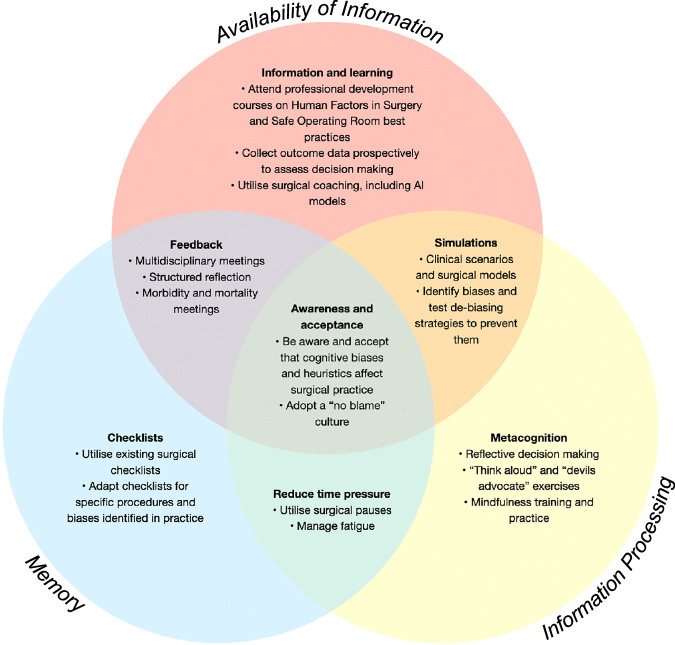
Practical recommendations to mitigate cognitive biases and heuristics in surgical settings by bias category.^43,44,55,56^

Practical recommendations, adapted from research in medical settings,^43,44,55,56^ to help mitigate cognitive biases and heuristics in surgical settings, are available in Figure 5.

### Strengths and Limitations

The strength of this review is the comprehensive search of 5 databases. Broad search terms were employed to compensate for the lack of taxonomy of biases and, therefore, the wide range of terminology employed in the literature, which resulted in 20,520 papers being screened. We were, however, unable to perform comparative statistical analyses or meta-analysis due to the heterogeneity of reporting in the identified studies.

## CONCLUSIONS

Our systematic review found that cognitive biases and heuristics consistently impacted surgeon’s decision making in preoperative, intraoperative, and postoperative settings. Anchoring and overconfidence bias were most likely to result in fatal outcomes. The development and incorporation of debiasing strategies within routine surgical training is, therefore, essential. Surgeons should also consider expanding, validating, and standardizing current surgical checklists with implementation at all stages of surgical care.

## Supplementary Material
