# Supplementary material for: Cognitive Biases and Heuristics in Surgical Settings: A Systematic Review
Source: Ann Surg. 2025 Apr 21;282(6):946–53. doi: 10.1097/SLA.0000000000006736 (PMC12594140; doi:10.1097/SLA.0000000000006736)
Supplement: Supplementary file 1 [file sla-282-0946-s001.docx]

**Supplemental Digital Content**

**Table 1: Included Study Characteristics**

| **Author** | **Year** | **Sample*** | **Speciality** | **Design** | **Aim** | **Identified Biases** | **Bias Testing** | **Key Findings** |
| --- | --- | --- | --- | --- | --- | --- | --- | --- |
| Antonacci[^20^](https://www.zotero.org/google-docs/?2SDo8e) | 2021 | 655 cases | General | Prospective Cohort | To evaluate the presence or absence of cognitive biases in patients sustaining a range of post-operative complications. | Aggregate bias, anchoring, ascertainment bias, commission bias, confirmation bias, diagnosis momentum, hindsight bias, omission bias, order effects, outcome bias, overconfidence bias, posterior probability error, premature closure, psych-out error, search satisfying, sunk cost fallacy, sutton's slip, triage cueing, unpacking principle, vertical line failure, visceral bias, yin-yang out | Biases identified by surgeons or trained evaluators: residents evaluated whether a bias affected the management of cases, evaluation quality was supervised by a surgical attending not involved in care management. | When a bias is present, complications are more serious in 76% of cases (*p* = .011), and serious complications occur 1.59 times (or 60%) more frequently when a bias is identified. |
| Antonacci[^21^](https://www.zotero.org/google-docs/?SMU6xh) | 2021 | 736 cases | General | Prospective Cohort | To report the incidence and distribution of cognitive biases, and evaluate their impact on management errors and standard of care. | Aggregate bias, anchoring, ascertainment bias, commission bias, confirmation bias, diagnosis momentum, hindsight bias, omission bias, order effects, outcome bias, overconfidence bias, posterior probability error, premature closure, psych-out error, search satisfying, sunk cost fallacy, suttons slip, triage cueing, unpacking principle, vertical line failure, visceral bias, yin-yang out | Biases identified by surgeons or trained evaluators: residents were trained in the definitions and clinical relevance of 22 biases and recorded evaluations of biases on cases with complications which were corrected by a supervising attending surgeon. | Presence of a cognitive bias was correlated with an increase in management, diagnostic, judgement, communication, therapeutic, and professionalism errors (*p* < .0001). Biases were related to significant decreases in the standard of care being met (*p* < .0001), increases in the standard of care having opportunities for improvement (*p* < .0001), and increases in the standard of care not being met (*p* < .0001). |
| Chaves[^37^](https://www.zotero.org/google-docs/?5iY9al) | 2022 | 55 surgeons  (27 allocated to deliberate reflection, 28 allocated to control group) | Orthopaedics | Controlled experimental study | To examine whether deliberate reflection reduces confirmation bias and increases diagnostic accuracy. | Confirmation bias | Objective measure: confirmation bias score based on the sum of confirmations for each group (control and experimental) in cases in which the wrong referral diagnosis was chosen. | Reduction in the presence of confirmation bias between the non-deliberate reflection and deliberate reflection task was only statistically significant in the experimental group (*p* = .003). |
| Garcia-Retamero[^26^](https://www.zotero.org/google-docs/?ofXL0I) | 2019 | 300 surgeons | Trauma, craniomaxillofacial, spine, and others | Two part computer-based questionnaire | Primary aim: to investigate whether reporting results in absolute numbers versus percentages in groups of patients with varying denominators would influence the accuracy of surgeons’ estimations of risk reduction and to assess their perceptions of clarity of the provided information.  Secondary aim: to investigate whether adding percentages to absolute numbers would improve the accuracy of estimations of risk reduction and to assess the extent to which this would increase associated perceptions of clarity. | Denominator neglect | Objective measure: response latency in risk reduction, response latency in perceptions of clarity of information. | When numbers were reported without denominators or with different denominators than when reported with the same denominators, surgeons perceived the risk reduction to be larger (*p* < .001). Risk reduction was perceived to be larger with absolute numbers when reported without denominators than with different denominators (*p* = .003). |
| Hughes-Hallet[^27^](https://www.zotero.org/google-docs/?7sUsN7) | 2015 | 73 surgeons  (34 allocated to the low cognitive load group, 39 to the high cognitive load group; then 24 allocated the no image guidance group, and the remaining 49 divided into 24 in the wireframe overlay group, and 25 in the solive overlay group | Laparoscopic partial nephrectomy | Randomised control | To determine the effect of cognitive load and surgical image guidance on operative inattention blindness. | Inattention blindness | Objective measure: counting based tasks, a computer-based tool to assess the degree of inattention blindness, and the NASA-TLX to assess cognitive load. | The level of prompted inattention blindness was 74%. The high cognitive load group had a significantly higher NASA-TLX score (*p* = .04) and significantly higher inattention blindness (*p* = .002). |
| Janssen[^22^](https://www.zotero.org/google-docs/?2mvu8f) | 2021 | 196 surgeons | Orthopaedics | Survey | To measure the prevalence of four biases in patient vignettes. | Anchoring, base rate neglect, framing, confirmation bias | Author developed survey: base rate neglect was evaluated by checking if answers to three scenarios considered the base rate; incorrect answers ignored it. Confirmation bias was assessed with five scenarios, marking answers that showed bias as incorrect. Anchoring was tested by giving participants different estimates, high or low anchors, (79% or 31%) for a patient’s arthritis risk and comparing their predictions. Framing effect was measured by presenting scenarios about an antibiotic with positive or negative descriptions and noting if participants preferred the antibiotic more after the positive frame, despite both descriptions being mathematically equivalent. | Overall incidence of base rate neglect = 43% and of confirmation bias - 51%. The clinical vignette with the higher anchor had a significantly higher estimate of probability of developing osteoarthritis (*p* < .001). The emphasis on positive framing of an antibiotic prescription results in significantly higher likelihood of prescription (*p* = .002). |
| Karnick[^38^](https://www.zotero.org/google-docs/?bfsfKP) | 2021 | 34 surgeons | General surgery | Experiment and survey | To assess the presence of self- evaluation bias in a cohort of residents. | Self-evaluation bias | Objective measure: bias was measured by the difference between estimated and actual scores of competence. | Surgeons actual performance scores and self-assessments were positively correlated (*p* < .001). Self-estimates were lower than actual performance (*p* = .002). Residents with higher performance scores were less accurate at self-estimating (*p* = .009). Surgeons who were more accurate at estimating their competence showed greater improvement at the end of the year (*p* = .018). |
| Katt[^28^](https://www.zotero.org/google-docs/?n1HRvC) | 2021 | 9 surgeons | Orthopaedics | Self-administered questionnaire | Primary aim: to determine how accurately surgeons’ estimate time needed to complete procedures.  Secondary aim: to determine how accuracy of surgical time estimations correlated with increased repetition and site location of service, and how accuracy of estimations varied with increased surgical durations. | Planning fallacy, optimism bias | Objective measure: Bias was measured by the difference between absolute and estimated operative time and total room time. | Average estimated total room time was 78.2 minutes and actual total room time was 82.7 minutes (*p* = .034). All surgeons showed a decrease in average absolute difference between estimated operative time and actual operative time (*p* = .031). |
| MacDermid[^29^](https://www.zotero.org/google-docs/?sD5dZ7) | 2014 | 110 surgeons | Rectal | Self-administered questionnaire | To identify patient and surgeon factors which would influence the decision to defunction a range of hypothetical  rectal anastomoses. | Risk aversion | Validated survey: survey on risk-taking and a bias score derived from hypothetical patient scenarios. | Age (*p* = .0379) and total risk-taking score (*p* = .0407 were found to exert an effect on the decision to create a stoma after anterior resection. |
| MacDermid[^30^](https://www.zotero.org/google-docs/?w6Fx5q) | 2017 | 150 surgeons | Rectal | Self-administered questionnaire | To assess age and risk-taking propensity and identify other potential biases. | Risk aversion | Validated survey: survey on risk-taking and a bias score derived from hypothetical patient scenarios. | Age (*p* = .02), total risk score (*p* = .044), belief that their own leak rate was lower than average (*p* = .02), and belief that the average risk of anastomotic leak after low anterior resection was ≤8% (*p* = .007) were found to exert an effect on decision to create defunctioning stomas. |
| Malhotra[^31^](https://www.zotero.org/google-docs/?8tfDsP) | 2012 | 31 surgeons | Hepatobiliary and pancreatic, colorectal, esophageal and  upper gastrointestinal | Simulated scenario in a laparoscopic environment | To investigate error management  by novices compared with experienced surgeons when performing a simple simulated incision in a visually challenging  environment. | Perceptual bias | Objective measure: computed percentage difference between absolute and estimated incision line. | Significant main effect of visual feedback in the perceptual illusion condition, with underestimations more pronounced when no visual feedback was available (*p* < .001). |
| Pandit[^32^](https://www.zotero.org/google-docs/?erxpx8) | 2022 | 28 surgeons  (13 allocated to intervention group, 15 to control group) | Neurosurgery | Prospective controlled pilot | To determine if a mindfulness-based intervention reduced inattention blindness. | Inattention blindness | Objective measure: calculation of a median absolute error score for attention and inattention tasks to reflect the difference between the perceived and actual counts of surgical instruments in the operative field. | The mindfulness intervention group had lower inattention scores than the control group (*p* - .02). Inattention scores differed significantly between the first and second operative videos for both groups (*p* < .0001) with inattention errors reduced by -1.64 for the second video. |
| Redelmeier[^33^](https://www.zotero.org/google-docs/?hbJ8G4) | 1995 | 352 neurologists and neurosurgeons | Neurosurgery | Survey | To determine whether situations involving multiple options can paradoxically influence people to choose an option that would have been declined if fewer options were available. | Framing | Author developed survey: framing effect was identified when surgeons chose to prioritise one patient from a selection of three patients and to not prioritise the same patient in a selection of two patients. | When asked to decide to prioritise patients for carotid artery surgery, surgeons avoided deciding between two similar patients and instead decided on an alternative, entirely clinically different patient (*p* < .001). |
| Sacks[^34^](https://www.zotero.org/google-docs/?yV3RCt) | 2021 | 1769 surgeons | General | Survey | To assess whether risk aversion influences surgical decisions and whether the relationship can be explained by differences in perception of treatment risks and benefits. | Risk aversion | Validated survey: six item risk aversion survey modified by adding “in my surgical practice” to each item. | Significant association was found between risk aversion and the decision to recommend the operation with risk-averse surgeons less likely to recommend an operation (*p* = .018). More risk averse surgeons perceived a higher probability of surgical complications (*p* =.01). |
| Simianu[^35^](https://www.zotero.org/google-docs/?CZisQT) | 2016 | 282 surgeons | Colorectal | Prospective cohort | To assess recency effect by examining the use of preventative leak testing before and after colorectal operations with anastomotic leaks. | Recency bias | Objective measure: recency bias was assessed through measuring change in the rate in anastomotic leak testing performed by each surgeon. A recency effect was identified when a clinician increased leak testing rates by at least 5% in the prior 6 months. | Recency bias was found in one-third of surgeons, with surgeons demonstrating recency bias more frequently performing operations for diverticulitis (*p* < .001), more frequently beginning their cases minimally invasively (*p* < .001), and having longer mean operative times (*p* <.001). |
| Tait[^39^](https://www.zotero.org/google-docs/?ZFyF1P) | 2005 | 40 surgeons | Neurosurgery and orthopaedics | Survey | To examine attributions for treatment success or failure as moderated by the role of empathy. | Self-serving attributional bias | Author developed survey: four clinical vignettes designed to assess attributions of patient’s surgical outcome to physical skill, psychological factors and both. | Surgeons were more likely to make attributions to physical factors, such as pain severity and patient disability, for the surgical outcome when conservative treatment was successful (*p* <.01). Surgeons were more likely to make attributions to psychological factors, such as psychiatric distress and patient effort, for outcomes when treatment was unsuccessful (*p* <.001). |
| Teunis[^25^](https://www.zotero.org/google-docs/?FS5E78) | 2016 | 242 surgeons | Orthopaedics | Survey | Primary aim: to assess whether uncertainty and overconfidence bias decrease with years of practice.  Secondary aim: to assess if overconfidence bias, degree of trust in evidence, and degree of statistical sophistication correlate independently with recognition of uncertainty. | Overconfidence bias | Author developed survey: reviewed by the American Association for Hand Surgery Research Listserv and evaluated for internal consistency using the Cronbach alpha of each domain. | Overconfidence bias increased by years of practice (*p* <.001). Greater overconfidence bias was correlated with less recognition of uncertainty (*p* <.001). Greater trust in the orthopaedic evidence base was correlated with less recognition of uncertainty (*p* = .002). Better statistical understanding was correlated with greater recognition of uncertainty (*p* <.001). |
| Thiels[^40^](https://www.zotero.org/google-docs/?bm6qZM) | 2015 | 1.5 million cases | Unspecified | Prospective analysis | To analyse human factors, including cognitive biases, contributing to invasive procedural never events. | Confirmation bias | Validated tool: trained quality specialist coded each error-contributing category e.g., confirmation bias using a validated tool: the Human Factors Taxonomy for Healthcare. | Half of the contributing human factors to 69 never events that underwent root cause analysis were due to cognitive biases. Confirmation bias was the most common (*n* = 36). |
| Vogel[^24^](https://www.zotero.org/google-docs/?CXBQ9R) | 2019 | 230 cases | Colorectal | Structured interview & structured morbidity and mortality conferences | To investigate the effect of errors due to cognitive heuristics during the course of surgical treatment. | Anchoring, availability bias, commission bias, overconfidence bias, omission bias, sunk cost fallacy | Biases identified by surgeons or trained evaluators: cognitive biases were identified by independent coders who watched structured interviews with each surgeon. | Cognitive biases were found in 7/8 investigated procedures in patients with anastomotic failure after colorectal resection: anchoring (*n* = 1), availability bias (*n* = 1), commission bias (*n* = 1), overconfidence bias (*n* = 1), omission bias (*n* = 2), and sunk cost fallacy (*n* = 1). |
| Whelehan[^23^](https://www.zotero.org/google-docs/?uGv4Cq) | 2021 | 73 surgeons | General | Cross sectional survey | To explore how surgeons make decisions in high-stake biliary tract clinical scenarios. | Anchoring, availability bias, confirmation bias | Author-developed survey: clinical vignette survey modelled on the theories of Tversky and Kahneman that was piloted prior to use. | Of surgeons provided with an anchor, 71.4% chose to refer the patient to a consultant. Availability bias, influenced by an "availability hook" (a specific example or piece of information that makes certain outcomes seem more likely), led to increased premature confidence in a diagnosis, with 80% of those not given this hook choosing to consult another surgeon. Confirmation bias, where existing beliefs influenced decision-making, led to 78.6% of surgeons opting to stop a procedure and conduct postoperative imaging. |
| Whyte[^36^](https://www.zotero.org/google-docs/?Z12PJS) | 2022 | 51 surgeons | Plastic, breast, endocrine | Survey | To explore how seven cognitive biases affect restorative surgery decision-making in elective settings | Confirmation bias, endowment effect, framing, herding bias, illusion of control, risk aversion, representation heuristic | Author-developed survey: covering seven cognitive biases. | All seven biases were identified in surgeons: representation heuristic in 67.31%, framing in 27.45%, illusion of control in 41.18%, endowment effect in 47.06%, herding bias in 76.47%, confirmation bias in 31.37%, and two types of risk aversion in 76.47% and 43.15%. |

*** Studies included in this review that include a patient population either report the number of “patients” or “cases”. For clarity, all patients are referred to as cases in this table.**

**Table 2: Identified Cognitive Biases Grouped into Three Bias Categories**

| **Cognitive Bias or Heuristic** | **Bias Category** | **Definition** | **Surgical example** |
| --- | --- | --- | --- |
| Aggregate Bias | IP | Occurs when multiple pieces of information are combined in a way that leads to mistaken conclusions as each individual piece was inaccurate or skewed. | *Combining recovery data from diverse hospitals misleads surgeons about expected patient outcomes, for example merging recovery rates from different trusts results in false expectations of recovery.* |
| Anchoring Bias | AoI | Occurs when individuals rely on the initial piece information (the ‘anchor’) they receive when making decisions to guide all future decisions, regardless of how misleading, inadequate or irrelevant this information is. | *A surgeon focuses on initial symptoms, leading to misdiagnosis if subsequent information isn't thoroughly considered and used for re-evaluation.* |
| Ascertainment Bias | AoI | The systematic distortion of certain groups or outcome measures in a sample due to errors in sampling and/or data collection. | *Only patients with easily detectable symptoms are considered for a specific treatment, those with more complex symptoms are overlooked for the procedure.* |
| Availability Bias | AoI | The tendency to rely on information that is easily available and mentally accessible leading to an overestimation of the importance of this data. | *A surgeon is more concerned about a complication they recently saw, neglecting other potential risks.* |
| Base Rate Neglect | AoI | The tendency to overlook the true prevalence or “base rate” of an outcome in favour of current, case-specific information. | *A surgeon underestimates the risk of a common complication because they recently had a successful outcome.* |
| Commission Bias | IP | The inclination towards action versus inaction when decision making, despite evidence that inaction may be more beneficial. | *A surgeon chooses to operate despite evidence suggesting non-surgical surveillance treatment may be safer.* |
| Confirmation Bias | Memory | When individuals seek, focus on and retain information that aligns with pre-existing beliefs. | *A surgeon interprets the patients’ history to support their initial diagnosis, overlooking contradictory information that suggests a different condition.* |
| Denominator Neglect | IP | When individuals focus solely on the number of events that occur (absolute number) and ignore how many chances there were for those events to happen (total number). | *A surgeon only considers the number of successful surgeries performed, ignoring the total number of surgeries conducted, when assessing the procedure’s safety.* |
| Diagnosis Momentum | IP | Occurs when an initial diagnostic label becomes increasingly entrenched over time, transitioning from possibilities to certainties through communication between patients, nurses, and physicians. | *A patient initially given a suspected diagnosis by a clinician that has not been fully investigated or confirmed repeats this diagnosis and certain during a surgical consultation. Over repeat consultations, alternative diagnoses are not considered.* |
| Endowment Effect Bias | IP | Where individuals are more likely to irrationally overvalue an object because they own it, as opposed to recognising its true market value. | *A surgeon overvalues a technique they are accustomed to and that they planned for, disregarding potentially more effective methods.* |
| Framing Effect | IP | Occurs when decision making is influenced by the way in which information is presented. | *A surgeon perceives a procedure as riskier when it is presented as having a “15% failure rate” versus a “85% success rate”.* |
| Herding Bias | IP | Where individuals are more likely to make decisions based on the actions taken by others in a group than their own judgement or research. | *A surgeon adopts a popular surgical technique because colleagues use it, without considering its suitability for each individual patient.* |
| Hindsight Bias | Memory | When past events are perceived as having been accurately predicted beforehand, despite the unpredictable nature of the event. | *Following an unexpected complication a surgeon wrongly believes they predicted it overlooking the known unpredictable nature of the surgery, falsely attributing the outcome to their own foresight.* |
| Illusion of Control | IP | Occurs when individuals overestimate the level of control they have over the outcome of an event. | *A surgeon believes their actions alone determine a patient’s outcome, disregarding all other factors.* |
| Inattention Blindness | IP | The inability to recognise objects within one’s visual field due to diverted attention elsewhere. | *A surgeon does not notice a small bleed because their focus is on resection of the operative lesion* |
| Omission Bias | IP | The tendency to judge harmful actions more negatively than equally harmful inactions, despite the same resulting outcome. | *A surgeon avoids recommending surgery, despite its potential benefit, to prevent complications and subsequent blame if they occur.* |
| Optimism Bias | IP | The tendency for individuals to underestimate their chances of negative outcomes and overestimate their chances of positive outcomes, leading to overly optimistic predictions and decisions. | *A surgeon believes a complex surgery will have a smoother recovery than statistically likely, underestimating potential complications.* |
| Order Effects | AoI | Refers to how the position and sequence of tasks presented to individuals can influence decision making, resulting in various outcomes based entirely on the sequence of presentation. | *When discussing treatment options with colleagues, a surgeon feels more confident about a procedure that is presented first.* |
| Outcome Bias | IP | Occurs when healthcare professionals choose a treatment because it might lead to a better patient outcome, even though it is less likely to succeed, instead of opting for a more reliable but less optimal treatment. | *A surgeon chooses a high risk and unpredictable surgery which, if successful, will result in an optimal outcome although the chance of this occurring is low, instead of a lower risk procedure that may predictably leave minor postoperative deficits.* |
| Overconfidence Bias | AoI | A tendency to overestimate one’s knowledge and abilities, potentially leading to poor decision making. | *A surgeon attempts a complex procedure without adequate experience, overestimating their ability to handle potential complications.* |
| Perceptual Bias | IP | The tendency for individuals to misinterpret sensory information, particularly visual information, leading to an erroneous perception of reality. | *During surgery, a surgeon perceives an incision to be longer or shorter than it actually is, leading to the inaccurate execution of the procedure.* |
| Planning Fallacy | IP | When individuals tend to underestimate the time, costs, and risks of future actions or projects despite past experience and evidence. | *A surgeon underestimates the time needed for a surgery, leading to delays and issues with subsequent procedures in the theatre list* |
| Posterior Probability Error | Memory | Occurs when individuals are disproportionately affected by past events, preventing prior beliefs from being updated despite contradictory or new evidence. | *A surgeon persists with a procedure despite new research indicating its ineffectiveness due to their own past success with the method.* |
| Premature Closure | AoI | Occurs in clinical practice when individuals prematurely reach a diagnosis without adequately exploring other possible diagnoses and collecting supporting evidence. | *A surgeon quickly concludes a diagnosis without considering other possibilities, potentially leading to missed or incorrect treatment.* |
| Psych-Out Error | AoI | Where healthcare professions may assume a patient has a psychiatric diagnosis and therefore overlook an underlying physiological health issue. | *A surgeon overlooks a patient's complaints of pain, assuming they are psychosomatic, delaying diagnosis of a surgical complication.* |
| Recency Bias | IP | When individuals give more weight to the most recent information or events when making decisions or judgements, often overlooking older or historical data. | *A surgeon prioritises a recent study's findings over older, well-established research when deciding on a surgical technique.* |
| Representation Heuristic | Memory | Occurs when individuals estimate the likelihood of an event occurring based on how similar it is to prior events or familiar examples despite more relevant information. | *A surgeon assumes a patient's symptoms indicate a common condition they've seen before, overlooking rare possibilities.* |
| Risk Aversion | IP | The tendency to favour outcomes with low uncertainty and avoid high uncertainty scenarios, even if the latter is higher-yield. | *A surgeon chooses a less effective but well-established technique over a riskier but potentially more beneficial procedure.* |
| Search Satisfying | AoI | Occurs when all investigations for a patient are stopped once the first plausible diagnosis is found. | *Upon identifying a potential cause for a patient's pain, a surgeon forgoes additional tests, overlooking underlying complications.* |
| Self-Evaluation Bias | Memory | When individuals assess their own performance, abilities, or characteristics in a way that over or under estimates their actual ability, likely due to limited self-awareness. | *Following a procedure, a surgeon overlooks their own mistakes and personal factors that led to operative complications.* |
| Self Serving Attributional Bias | Memory | The tendency to credit positive outcomes with internal factors (e.g., intelligence) and negative outcomes due to external factors (e.g., poor luck) resulting in a positive self image that does not reflect actual ability. | *A surgeon attributes successful surgeries to their skill but blames complications on external factors such as patient condition or equipment.* |
| Sunk Cost Fallacy | IP | Occurs when individuals continue to invest time, effort, or money into a decision and are reluctant to abandon it, despite evidence that this would be more valuable. | *A surgeon persists with a failing surgical approach to avoid admitting initial investment (time, effort) was wasted.* |
| Suttons Slip | IP | Occurs when all possibilities in a given scenario are not properly considered with the most obvious (e.g., most urgent or fastest outcome) given the majority, if not all of the focus. | *During emergency surgery, a surgeon prioritises addressing the most urgent issue, overlooking other critical factors.* |
| Triage Cueing | AoI | A predisposition towards a diagnosis made by a triage physician who may not have had adequate time to take a detailed history. As a result, outlier symptoms or underlying conditions which do not comply with this diagnosis may be ignored. | *In the emergency room, a patient complaining of chest pain is quickly referred to a cardiothoracic surgeon rather than a generalist physician for evaluation, influencing subsequent treatment decisions towards surgical interventions.* |
| Unpacking Principle | AoI | Failure to elicit all relevant information when establishing a diagnosis can lead to other possibilities being overlooked. | *A surgeon overlooks a patient's history of prior surgeries, leading to missed complications and technical difficulties during the current operation.* |
| Vertical Line Failure | IP | The tendency to fall into predictable, inflexible thought patterns when doing routine, repetitive tasks which emphasise efficacy and utility. | *During surgery, a surgeon follows the same steps for a routine procedure without adapting to unexpected complications.* |
| Visceral Bias | Memory | The influence of affect on decision making, often leading to poor decisions. | *During surgery, a surgeon hesitates to perform a necessary but risky procedure due to fear of negative emotions such as guilt.* |
| Yin-Yang Out | AoI | The tendency to believe that after comprehensive investigations which have yielded negative results, no further diagnostic effort will be fruitful and therefore no further investigations are carried out to determine an underlying diagnosis. | *Despite negative or inconclusive tests, a surgeon halts further investigations, assuming no additional findings are possible, overlooking important diagnoses.* |

**Search strategies**

**Medline (Ovid)**

1. exp Specialities, Surgical
2. exp Elective Surgical Procedures/ or exp Prophylactic Surgical Procedures/ or exp Cytoreduction Surgical Procedures/ or exp Ophthalmologic Surgical Procedures/ or exp Vascular Surgical Procedures/ or exp Reconstructive Surgical Procedures/ or exp Oral Surgical Procedures/ or exp Ambulatory Surgical Procedures/ or exp Surgical Oncology/ or exp Nasal Surgical Procedures/ or exp Robotic Surgical Procedures/ or exp Urogenital Surgical Procedures/ or exp Urologic Surgical Procedures/ or exp Sutureless Surgical Procedures/ or exp Biliary Tract Surgical Procedures/ or exp Dermatologic Surgical Procedures/ or exp Endocrine Surgical Procedures/ or exp Urologic Surgical Procedures, Male/ or exp Gynecologic Surgical Procedures/ or exp Minimally Invasive Surgical Procedures/ or exp Otologic Surgical Procedures/ or exp Ultrasonic Surgical Procedures/ or exp Thoracic Surgical Procedures/ or exp Digestive System Surgical Procedures/ or exp Orthognathic Surgical Procedures/ or exp Surgical Procedures, Operative/ or exp Cardiac Surgical Procedures/
3. exp Surgeons
4. surg*.mp
5. 1 or 2 or 3 or 4
6. exp bias
7. exp Heuristics
8. (cognit* adj3 bias*).tw.
9. (operative adj3 bias*).tw.
10. heuristic*.tw.
11. 6 or 7 or 8 or 9 or 10
12. 5 and 11

**Embase (Ovid)**

1. exp Specialities, Surgical
2. exp Elective Surgical Procedures/ or exp Prophylactic Surgical Procedures/ or exp Cytoreduction Surgical Procedures/ or exp Ophthalmologic Surgical Procedures/ or exp Vascular Surgical Procedures/ or exp Reconstructive Surgical Procedures/ or exp Oral Surgical Procedures/ or exp Ambulatory Surgical Procedures/ or exp Surgical Oncology/ or exp Nasal Surgical Procedures/ or exp Robotic Surgical Procedures/ or exp Urogenital Surgical Procedures/ or exp Urologic Surgical Procedures/ or exp Sutureless Surgical Procedures/ or exp Biliary Tract Surgical Procedures/ or exp Dermatologic Surgical Procedures/ or exp Endocrine Surgical Procedures/ or exp Urologic Surgical Procedures, Male/ or exp Gynecologic Surgical Procedures/ or exp Minimally Invasive Surgical Procedures/ or exp Otologic Surgical Procedures/ or exp Ultrasonic Surgical Procedures/ or exp Thoracic Surgical Procedures/ or exp Digestive System Surgical Procedures/ or exp Orthognathic Surgical Procedures/ or exp Surgical Procedures, Operative/ or exp Cardiac Surgical Procedures/
3. exp Surgeons
4. surg*.mp
5. 1 or 2 or 3 or 4
6. exp bias
7. exp Heuristics
8. (cognit* adj3 bias*).tw.
9. (operative adj3 bias*).tw.
10. heuristic*.tw.
11. 6 or 7 or 8 or 9 or 10

**PsycINFO (Ovid)**

1. exp Surgery
2. exp Plastic Surgery/ or exp Bariatric Surgery/ or exp Heart Surgery/ or exp Endocrine Gland Surgery/
3. exp Surgeons
4. surg*.mp
5. 1 or 2 or 3 or 4
6. [bias.mp](http://bias.mp/)
7. exp Attentional Bias or exp Cognitive Bias or exp Hindsight Bias or exp Implicit Bias or exp Interpretive Bias or exp Response Bias or exp Test Bias
8. exp Heuristics
9. (cognit* adj3 bias*).ti,ab
10. (operative adj3 bias*).ti,ab
11. heuristic*.ti,ab
12. 6 or 7 or 8 or 9 or 10 or 11
13. 5 and 12

**SCOPUS**

INDEXTERMS ( *surgeon* ) OR INDEXTERMS ( *surgery* ) OR TITLE-ABS ( *surgeon** ) OR TITLE-ABS ( *surg** ) AND INDEXTERMS ( *bias* ) OR INDEXTERMS ( *heuristics* ) OR TITLE-ABS ( *cognit** W/3 *bias** ) OR TITLE-ABS ( *operative* W/3 *bias** ) OR TITLE-ABS ( *heuristic** )

**WoS**

(TS=Surgeon* OR TS=Surg*) AND (TI=(cognit* NEAR/3 bias*) OR AB=(cognit* NEAR/3 bias*) OR TI=(operative NEAR/3 bias*) OR AB=(operative NEAR/3 bias*) OR(TI=heuristic* OR AB=heuristic*))

**Data extraction form**

| **Category** | | **Answer** |
| --- | --- | --- |
| Descriptive Information | First author | Open answer |
|  | Year | Open answer |
|  | Title | Open answer |
|  | Journal | Open answer |
|  | Study aim | Open answer |
|  | Study design | From three categories: survey, analysis, experiment |
|  | Sample size | Open answer: number of surgeons, patients, or cases. |
| Surgery information | Speciality | Open answer |
|  | Surgical procedure | Open answer |
|  | Surgical outcomes measured | Open answer |
| Cognitive biases and heuristics investigations | Biases and/or heuristics investigated | Open answer |
|  | Prevalence | Open answer |
|  | Quantitative measures of effect | Open answer |
|  | Qualitative measures of effect | Open answer |
|  | Identified impact on surgery/surgeon/patient | Open answer |

**Included study funding information**

| **First Author** | **Year** | **Study Funding** |
| --- | --- | --- |
| Antonacci[^20^](https://www.zotero.org/google-docs/?4pMY7J) | 2021 | No funding, no funding agreements limiting the authors’ independence |
| Antonacci[^21^](https://www.zotero.org/google-docs/?xoGYyC) | 2021 | No funding, no funding agreements limiting the authors’ independence |
| Chaves[^37^](https://www.zotero.org/google-docs/?CRM7ZA) | 2022 | No funding, no funding agreements limiting the authors’ independence |
| Garcia-Retamero[^26^](https://www.zotero.org/google-docs/?KVjjAB) | 2019 | Funded by AO Foundation via AO Clinical Investigation and Documentation. Financial support provided by the Ministerio de Economia y Competitividad (Spain) and the National Science Foundation (United States). Authors declared independence from funding agencies. |
| Hughes-Hallet[^27^](https://www.zotero.org/google-docs/?fugoHF) | 2015 | No financial ties to disclose. |
| Janssen[^22^](https://www.zotero.org/google-docs/?qQbUWJ) | 2021 | No funding declared. |
| Karnick[^38^](https://www.zotero.org/google-docs/?mDUbY6) | 2021 | No external funding received for the project. |
| Katt[28](https://www.zotero.org/google-docs/?fHPUWB) | 2021 | No funding declared. |
| MacDermid[^29^](https://www.zotero.org/google-docs/?xWaLLV) | 2014 | Research grant from Sydney University. |
| MacDermid[^30^](https://www.zotero.org/google-docs/?RmQqbX) | 2017 | No funding declared. |
| Malhotra^31^ | 2012 | Bilateral research grant from the Economic and Social Research Council (UK) and the Research Grants Council (Hong Kong). |
| Pandit[^32^](https://www.zotero.org/google-docs/?cSdZFv) | 2022 | Supported by the National Brain Appeal’s Small Acorns Fund. |
| Redelmeier[^33^](https://www.zotero.org/google-docs/?I4L49y) | 1995 | Grant from the Physician’s Services Inc Foundation (Ontario, Canada) and a US Public Health Service grant from the National Institute of Mental Health (USA). |
| Sacks[^34^](https://www.zotero.org/google-docs/?HG8SZH) | 2021 | Financial support provided in part by The Robert Wood Johnson Clinical Scholars program. |
| Simianu[^35^](https://www.zotero.org/google-docs/?kzzuvZ) | 2016 | Supported by the National Institute of Diabetes and Digestive and Kidney Diseases of the National Institutes of Health and the University of Washington’s Department of Surgery Research Reinvestment Fund. |
| Tait[^39^](https://www.zotero.org/google-docs/?jgLOvZ) | 2005 | No funding declared. |
| Teunis[^25^](https://www.zotero.org/google-docs/?JR0l0r) | 2016 | No funding declared. |
| Thiels[^40^](https://www.zotero.org/google-docs/?MMCugI) | 2015 | Supported in part by the National Institute of Diabetes and Digestive and Kidney Diseases of the National Institutes of Health. |
| Vogel[^24^](https://www.zotero.org/google-docs/?9kGJ6M) | 2019 | No funding to declare. |
| Whelehan[^23^](https://www.zotero.org/google-docs/?w01OEv) | 2021 | Funding information is non-applicable. |
| Whyte[^36^](https://www.zotero.org/google-docs/?JDcQ8u) | 2022 | No external funding to declare. |

#

**PRISMA Checklist**

# PRISMA 2020 Main Checklist

| **Topic** | **No.** | **Item** | **Location where item is reported** |
| --- | --- | --- | --- |
| **TITLE** |  |  |  |
| **Title** | 1 | Identify the report as a systematic review. | Page 1 |
| **ABSTRACT** |  |  |  |
| **Abstract** | 2 | See the PRISMA 2020 for Abstracts checklist |  |
| **INTRODUCTION** |  |  |  |
| **Rationale** | 3 | Describe the rationale for the review in the context of existing knowledge. | Page 1 |
| **Objectives** | 4 | Provide an explicit statement of the objective(s) or question(s) the review addresses. | Page 1 |
| **METHODS** |  |  |  |
| **Eligibility criteria** | 5 | Specify the inclusion and exclusion criteria for the review and how studies were grouped for the syntheses. | Page 2 |
| **Information sources** | 6 | Specify all databases, registers, websites, organisations, reference lists and other sources searched or consulted to identify studies. Specify the date when each source was last searched or consulted. | Page 2 |
| **Search strategy** | 7 | Present the full search strategies for all databases, registers and websites, including any filters and limits used. | Page 2 & Supplementary Digital Content |
| **Selection process** | 8 | Specify the methods used to decide whether a study met the inclusion criteria of the review, including how many reviewers screened each record and each report retrieved, whether they worked independently, and if applicable, details of automation tools used in the process. | Pages 2 & 3 |
| **Data collection process** | 9 | Specify the methods used to collect data from reports, including how many reviewers collected data from each report, whether they worked independently, any processes for obtaining or confirming data from study investigators, and if applicable, details of automation tools used in the process. | Pages 2 & 3 |
| **Data items** | 10a | List and define all outcomes for which data were sought. Specify whether all results that were compatible with each outcome domain in each study were sought (e.g. for all measures, time points, analyses), and if not, the methods used to decide which results to collect. | Page 2 & Supplementary Digital Content |
|  | 10b | List and define all other variables for which data were sought (e.g. participant and intervention characteristics, funding sources). Describe any assumptions made about any missing or unclear information. | Page 2 |
| **Study risk of bias assessment** | 11 | Specify the methods used to assess risk of bias in the included studies, including details of the tool(s) used, how many reviewers assessed each study and whether they worked independently, and if applicable, details of automation tools used in the process. | Page 2 |
| **Effect measures** | 12 | Specify for each outcome the effect measure(s) (e.g. risk ratio, mean difference) used in the synthesis or presentation of results. | N/A |
| **Synthesis methods** | 13a | Describe the processes used to decide which studies were eligible for each synthesis (e.g. tabulating the study intervention characteristics and comparing against the planned groups for each synthesis (item 5)). | Page 2 & Supplementary Digital Content |
|  | 13b | Describe any methods required to prepare the data for presentation or synthesis, such as handling of missing summary statistics, or data conversions. | Page 2 |
|  | 13c | Describe any methods used to tabulate or visually display results of individual studies and syntheses. | Page 2 & Supplementary Digital Content |
|  | 13d | Describe any methods used to synthesize results and provide a rationale for the choice(s). If meta-analysis was performed, describe the model(s), method(s) to identify the presence and extent of statistical heterogeneity, and software package(s) used. | Page 2 & Supplementary Digital Content |
|  | 13e | Describe any methods used to explore possible causes of heterogeneity among study results (e.g. subgroup analysis, meta-regression). | N/A |
|  | 13f | Describe any sensitivity analyses conducted to assess robustness of the synthesized results. | N/A |
| **Reporting bias assessment** | 14 | Describe any methods used to assess risk of bias due to missing results in a synthesis (arising from reporting biases). | N/A |
| **Certainty assessment** | 15 | Describe any methods used to assess certainty (or confidence) in the body of evidence for an outcome. | N/A |
| **RESULTS** |  |  |  |
| **Study selection** | 16a | Describe the results of the search and selection process, from the number of records identified in the search to the number of studies included in the review, ideally using a flow diagram. | Page 2 & 3 |
|  | 16b | Cite studies that might appear to meet the inclusion criteria, but which were excluded, and explain why they were excluded. | N/A |
| **Study characteristics** | 17 | Cite each included study and present its characteristics. | Table 1 (Supplemental Digital Content) |
| **Risk of bias in studies** | 18 | Present assessments of risk of bias for each included study. | Figure 3 |
| **Results of individual studies** | 19 | For all outcomes, present, for each study: (a) summary statistics for each group (where appropriate) and (b) an effect estimate and its precision (e.g. confidence/credible interval), ideally using structured tables or plots. | Table 1 (Supplemental Digital Content) |
| **Results of syntheses** | 20a | For each synthesis, briefly summarise the characteristics and risk of bias among contributing studies. | Figure 2 & Page 3 |
|  | 20b | Present results of all statistical syntheses conducted. If meta-analysis was done, present for each the summary estimate and its precision (e.g. confidence/credible interval) and measures of statistical heterogeneity. If comparing groups, describe the direction of the effect. | N/A |
|  | 20c | Present results of all investigations of possible causes of heterogeneity among study results. | N/A |
|  | 20d | Present results of all sensitivity analyses conducted to assess the robustness of the synthesized results. | N/A |
| **Reporting biases** | 21 | Present assessments of risk of bias due to missing results (arising from reporting biases) for each synthesis assessed. | N/A |
| **Certainty of evidence** | 22 | Present assessments of certainty (or confidence) in the body of evidence for each outcome assessed. | N/A |
| **DISCUSSION** |  |  |  |
| **Discussion** | 23a | Provide a general interpretation of the results in the context of other evidence. | Pages 6 & 7 |
|  | 23b | Discuss any limitations of the evidence included in the review. | Page 6 & 7 |
|  | 23c | Discuss any limitations of the review processes used. | Page 7 |
|  | 23d | Discuss implications of the results for practice, policy, and future research. | Page 7 |
| **OTHER INFORMATION** |  |  |  |
| **Registration and protocol** | 24a | Provide registration information for the review, including register name and registration number, or state that the review was not registered. | Page 1 |
|  | 24b | Indicate where the review protocol can be accessed, or state that a protocol was not prepared. | Page 1 |
|  | 24c | Describe and explain any amendments to information provided at registration or in the protocol. | N/A |
| **Support** | 25 | Describe sources of financial or non-financial support for the review, and the role of the funders or sponsors in the review. | Page 1 |
| **Competing interests** | 26 | Declare any competing interests of review authors. | Page 1 |
| **Availability of data, code and other materials** | 27 | Report which of the following are publicly available and where they can be found: template data collection forms; data extracted from included studies; data used for all analyses; analytic code; any other materials used in the review. | N/A |

#####

# PRIMSA Abstract Checklist

| **Topic** | **No.** | **Item** | **Reported?** |
| --- | --- | --- | --- |
| **TITLE** |  |  |  |
| **Title** | 1 | Identify the report as a systematic review. | Yes |
| **BACKGROUND** |  |  |  |
| **Objectives** | 2 | Provide an explicit statement of the main objective(s) or question(s) the review addresses. | Yes |
| **METHODS** |  |  |  |
| **Eligibility criteria** | 3 | Specify the inclusion and exclusion criteria for the review. | Yes |
| **Information sources** | 4 | Specify the information sources (e.g. databases, registers) used to identify studies and the date when each was last searched. | Yes |
| **Risk of bias** | 5 | Specify the methods used to assess risk of bias in the included studies. | Yes |
| **Synthesis of results** | 6 | Specify the methods used to present and synthesize results. | Yes |
| **RESULTS** |  |  |  |
| **Included studies** | 7 | Give the total number of included studies and participants and summarise relevant characteristics of studies. | Yes |
| **Synthesis of results** | 8 | Present results for main outcomes, preferably indicating the number of included studies and participants for each. If meta-analysis was done, report the summary estimate and confidence/credible interval. If comparing groups, indicate the direction of the effect (i.e. which group is favoured). | Yes |
| **DISCUSSION** |  |  |  |
| **Limitations of evidence** | 9 | Provide a brief summary of the limitations of the evidence included in the review (e.g. study risk of bias, inconsistency and imprecision). | Yes |
| **Interpretation** | 10 | Provide a general interpretation of the results and important implications. | Yes |
| **OTHER** |  |  |  |
| **Funding** | 11 | Specify the primary source of funding for the review. | Yes |
| **Registration** | 12 | Provide the register name and registration number. | Yes |

*From:* Page MJ, McKenzie JE, Bossuyt PM, Boutron I, Hoffmann TC, Mulrow CD, et al. The PRISMA 2020 statement: an updated guideline for reporting systematic reviews. MetaArXiv. 2020, September 14. DOI: 10.31222/osf.io/v7gm2. For more information, visit: <www.prisma-statement.org>
